# Morphometric relationships between dimensions the anterior talofibular ligament and calcaneofibular ligament in routine magnetic resonance imaging

**DOI:** 10.1186/s40634-021-00406-2

**Published:** 2021-10-11

**Authors:** Pawel Szaro, Khaldun Ghali Gataa, Nektarios Solidakis, Przemysław Pękala

**Affiliations:** 1 Department of Radiology, Institute of Clinical Sciences, Sahlgrenska Academy, University of Gothenburg, Göteborgsvägen 31, 431 80 Gothenburg, Sweden; 2 Department of Musculoskeletal Radiology, Sahlgrenska University Hospital, Gothenburg, Sweden; 3 Department of Descriptive and Clinical Anatomy, Medical University of Warsaw, Warsaw, Poland; 4 Faculty of Medicine, Jagiellonian University Medical College, Krakow, Poland; 5 Faculty of Medicine and Health Sciences, Andrzej Frycz Modrzewski Kraków University, Kraków, Poland

**Keywords:** Ankle, Magnetic resonance imaging, Reconstruction, Graft, Double fascicular ligament

## Abstract

**Purpose:**

This study aimed to test the hypothesis that routine MRI ankle can be used to evaluate dimensions and correlations between dimensions of single and double fascicular variants of the ATFL and the CFL.

**Methods:**

We reviewed ankle MRIs for 251 patients. Differences between the length, thickness, width, and length of the bony attachments were evaluated twice. *P* < .05 was considered as significant.

**Results:**

For the ATFL, we observed a negative correlation between thickness and width, with a positive correlation between thickness and length (*p* < 0.001). The average values for the ATFL were thickness, 2.2 ± 0.05 mm; length, 21.5 ± 0.5 mm; and width, 7.6 ± 0.6 mm. The average values for the CFL were thickness, 2.1 ± 0.04 mm; length, 27.5 ± 0.5 mm; and width, 5.6 ± 0.3 mm. A negative correlation was found between length and width for the CFL (*p* < 0.001).

**Conclusions:**

Routine MRI showed that most dimensions of the ATFL and CFL correlate with each other, which should be considered when planning new reconstruction techniques and developing a virtual biomechanical model of the human foot.

**Level of evidence:**

III

## Background

The ankle instability and pain after surgical ligament reconstruction is seen in about 20% of patients, which requires further treatment [1]. Due to unsatisfactory clinical outcomes, there is a need to develop new reconstruction methods and to improve the traditional methods [2, 3]. The graft morphology and morphometry vary depending on the surgical technique but seem to be the most critical factors that influence the outcome of surgery [2]. To the best of our knowledge, there have been no studies on how the length, width, or thickness of the anterior talofibular ligament (ATFL) and the calcaneofibular ligament (CFL) correlate with each other in a normal in vivo MRI. Most studies have focused on only the length and width of these ligaments and have been performed on cadavers [4–7]. Therefore, comparing the results of these studies is challenging because the authors used different measurement techniques, thus obtaining noteworthy differences, even up to about 10 mm. Sometimes, it is unclear whether the measurements are for a single fascicular or double fascicular ligament. The length of the ATFL has been reported to vary from 12 mm [7] to 21 mm [4]. Based on the published literature, the length of the ATFL can be either 13–14 mm or 21–22 mm [4, 6], which may confirm that the values depend on the methodology and probably variations in fascicular structure. The length of the CFL is reported to be 15 mm [8] to 25 mm [5]. The width of the ATFL varies between studies from 6 mm [6] to 11 mm [8], while the width of the CFL varies from 5 mm [8] to 8 mm [5].

The MRI assessment of the thickness of the ATFL and the CFL is relevant for radiologists. The thickness may be a simple and objective factor that reflects the grade of ligament injury. The mean thickness of the ATFL reported in a previous anatomical study was 2.2 ± 0.6 mm, and the corresponding value for the CFL was 2.1 ± 0.5 mm [9]. However, there have been no radiological studies on larger samples. The MRI assessment of ATFL and CFL dimensions may be relevant for more tailored ankle modeling, virtual surgical planning, or mechanical interpretation of normal or impaired ankle status. Moreover, there is an ongoing discussion about how to reconstruct the internal structure of an injured ligament [10, 11], which is a similar discussion to what occurred for the anterior cruciate ligament double fascicular reconstruction in the past. Considering individual variability and the presence of connections between ligaments [12], finding a correlation between the dimensions of the ATFL and the CFL will enable the preparation of a more “custom-tailored” graft [5, 13, 14]. To the best of our knowledge, there have been no complex studies on measurements of the ATFL and the CFL and correlations in vivo, which was the aim of our study.

This study aimed to investigate the clinical anatomy of the ATFL and the CFL and their mutual relationship. The authors hypothesized that the thickness, length, and width of single and double fascicular variants of the ATFL and the CFL would correlate with each other.

## Methods

This was a retrospective single center study of 251 clinically indicated MRI scans of the ankle. Our study included 143 females and 108 males; the age range was 18–62 years, with a mean of 32.7 years. The right ankle was examined in 132 cases, and the left ankle was examined in 119 cases.

### Inclusion criteria

The MRI examinations were performed from January 2018 to May 2020 for non-traumatic indications. The imaging was performed on 1.5 Tesla (34 cases) and 3.0 Tesla (217 cases) MRI machine (Siemens and Philips). The inclusion criteria included use of a dedicated ankle coil and the availability of at least the following sequences: proton density (PD) or T2-weighted sequences without fat saturation in the sagittal, axial, and coronal planes to assess ligament structure. The other sequences, such as T1-weighted turbo spin echo (T1-TSE), PD with fat suppression, or short-T1 inversion recovery (STIR), were used to detect pathology. PD-weighted turbo spin echo (TSE): TE (the echo time) 45 ms, TR (the repetition time) 2800–5000 ms. T2-weighted (TSE) TE 60 ms, TR 3000–5000 ms. T1-weighted: TE 11.5 ms, TR 700–750 ms. Voxel 0.45 × 0.53 × 3.0 mm, slide thickness 3 mm, field of view (FOV) 14 cm. No interslice gap was in our protocol. A dedicated ankle coil was used for MRI acquisition.

### Repeatability of position

**Fig. 1:**
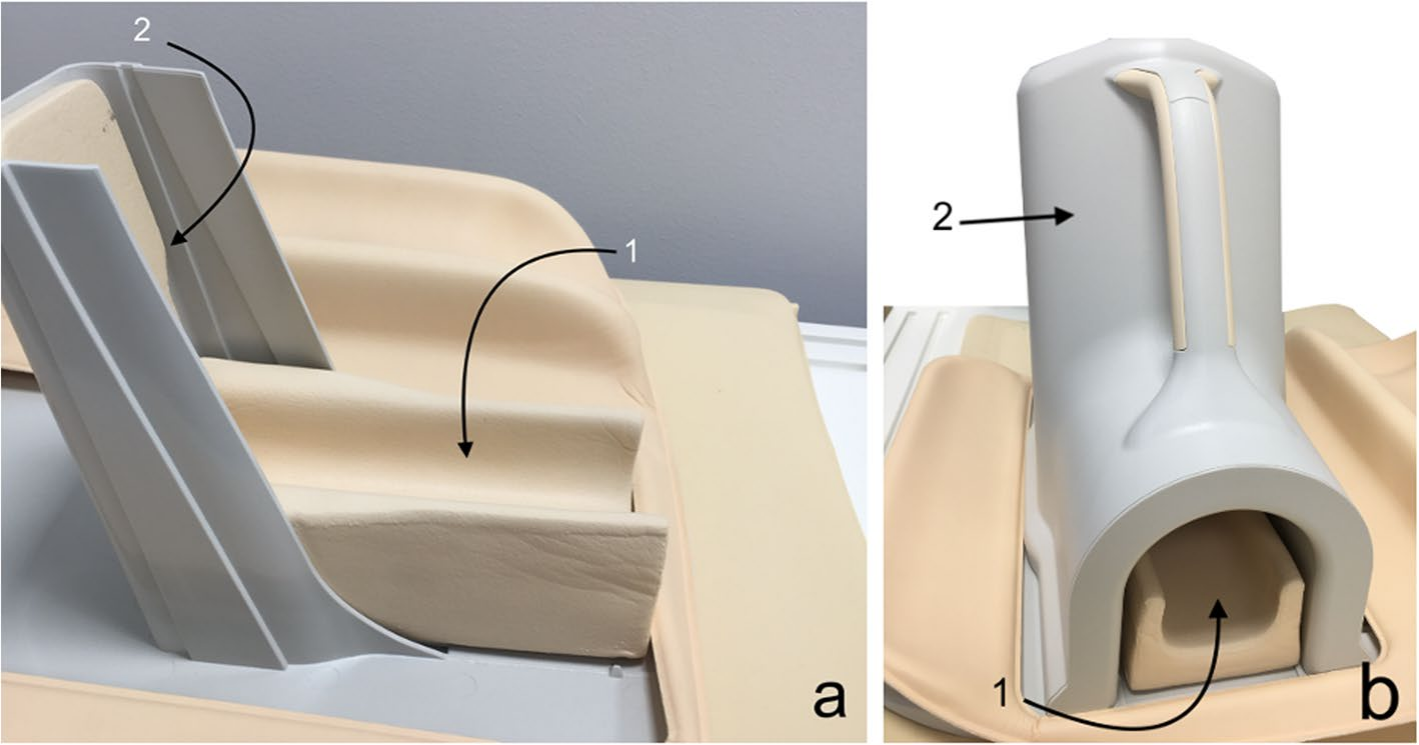
The coil is dedicated to the ankle and foot examination

The patient was in supine position and the location of the ankle joint was maintained by using a dedicated coil (Fig. 1) which was suited to the shape of the ankle and foot. Additional elastic wedge-shaped cushions were used to further secure the ankle and foot position.

### Evaluation of the ATFL and CFL

Detailed clinical evaluation of the ankle included in the referral letter. Uniform thickness, homogeneous signal, normal adjacent fat tissue, and no marrow edema or bone abnormalities indicate no trauma. MRI evaluation of the examined ligaments was done simultaneously by two experienced observers. Only normal ATFL and CFL were included in the study.

**Fig. 2:**
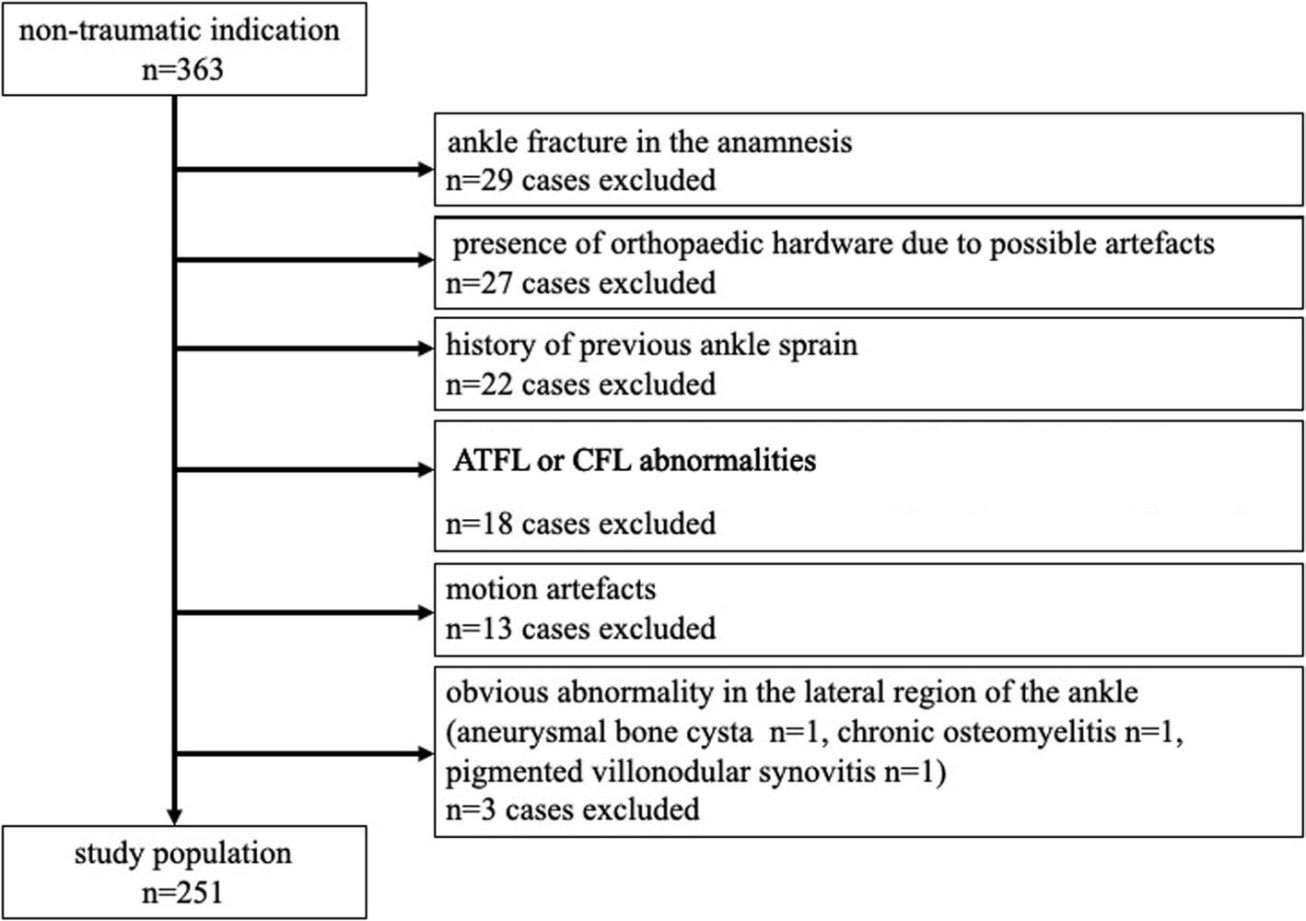
Study flow diagram

The exclusion criteria (Fig. 2) were ankle fracture in the anamnesis (29 cases excluded), the presence of orthopedic hardware due to possible artefacts (27 cases excluded), a history of previous ankle sprain (22 cases excluded), ATFL or CFL abnormalities (18 cases excluded), motion artefacts (13 cases excluded), and obvious abnormalities in the lateral region of the ankle (3 cases) (Fig. 2).

In total, 251 MRI examinations of the ankle fulfilled the inclusion and exclusion criteria (Fig. 2). All examinations were reviewed twice, and the final results were made by consensus.

### Nomenclature

We decided to use the term fascicule to define part of the ligament that is distinct and can be visualized on MRI. However, the appropriate nomenclature is missing regarding the internal ligament structure [13, 15].

### Measurement protocols

**Fig. 3:**
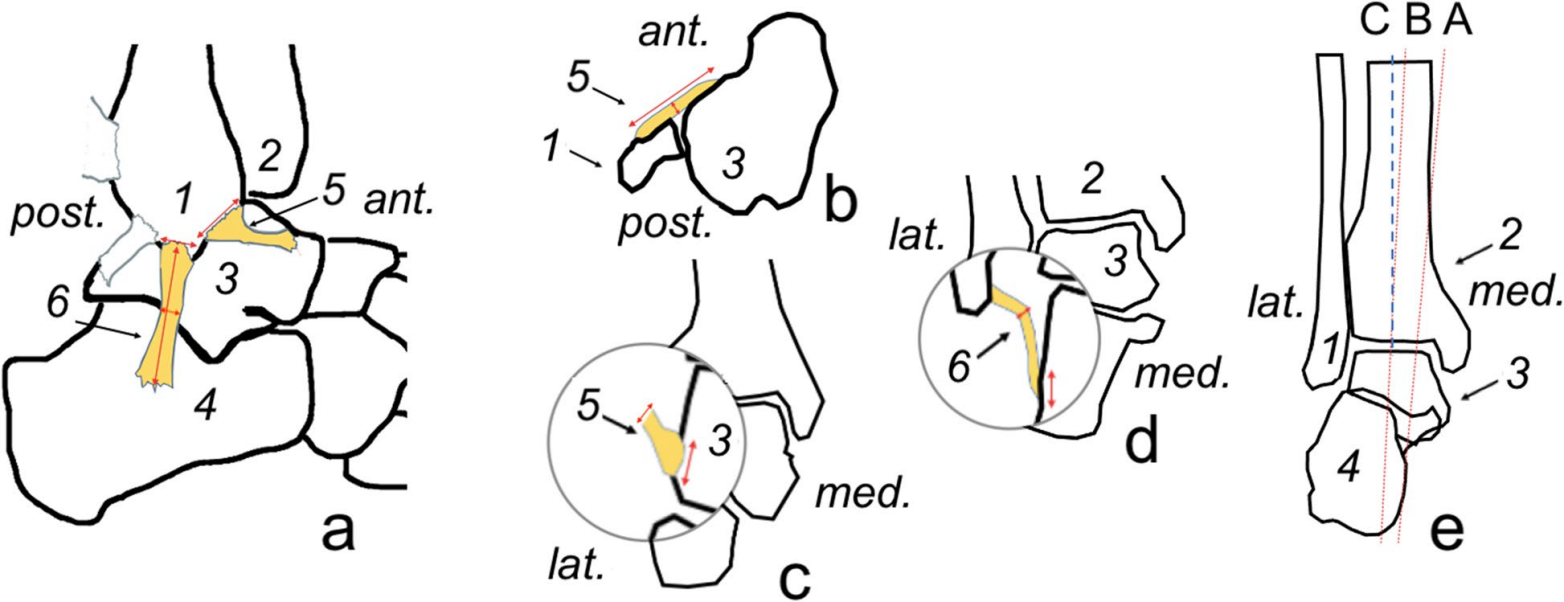
Method of measuring the anterior talofibular ligament and the calcaneofibular in our study. **A** Sagittal projection, view from the lateral side; **b** axial section; **c** and **d** coronal section. Ant., anterior; post., posterior; med., medial; lat., lateral; 1, the fibula; 2, the tibia; 3, the talus; 4, the calcaneus; 5, the ATFL; 6, the CFL

First, single, and multi-fascicular ligaments were identified. Then, measurements were assessed following the same protocol (Fig. 3). The approximate volume of the ATFL and the CFL was calculated using the cuboid formula. We used the following equation: volume of a cuboid = (length [mm] × width [mm] × thickness [mm]) mm^3^. Each MRI examination was reviewed twice by a musculoskeletal radiologist with 5 years of experience, who performed measurements twice with a minimum of a one-month interval. The mean value between two measurements was used for each subject. The inter-subject mean was used for the study. The other observer with 3 years of experience in musculoskeletal radiology performed measurements on one hundred and ten randomly chosen MRI examinations (alfa 0.05, Power 0.95) to evaluate the inter-observer agreement.

### Measurement of the ATFL

On the sagittal sequence, the length of the fibular attachment of the ATFL was measured (Fig. 3a). The length and thickness of the midportion of the ATFL was assessed on the axial sequence (Fig. 3b). The width of the ATFL was defined as the longest cranio-caudal dimension assessed in the midportion on the coronal section (Fig. 3c). On the coronal sequence, the largest dimension of the talar insertion was calculated (Fig. 3c).

### Measurement of the CFL

Consecutive sagittal slides were used to evaluate the CFL length and the width and length of the fibular insertion (Fig. 3a). The length of the calcaneal insertion was assessed on the coronal sequences (Fig. 3d). The thickness of the midportion of the CFL was assessed on the coronal sequence in the middle part of the ligament (Fig. 3d). In double fascicular ligaments, the width was measured for both fascicles together. The CFL angle and the tibiocalcaneal angle (TCA) were measured, as in a previous study [16]. Firstly, we chose the most posterior coronal section where both the tibia and calcaneus could be identified. Then, a line along the medial wall of the calcaneus was drawn (line A). Secondly, a coronal section that showed the maximum diameter of the tibial shaft was identified. The long axis of the tibial shaft was called the tibial shaft line (line B). The intersection angle between line B and the vertical axis (line C) was assessed to check if lines A and B were in the same direction or opposing directions. If lines A and B were in the same direction in relation to line C, then the TCA was between line A and B. If lines A and B were opposing in relation to line C, then the TCA was calculated by subtracting the angle between lines B and C from angle between lines A and C (Fig. 3).

Measurements were made at a dedicated diagnostic station using radiological software, with measures to 0.1 mm.

### Statistical analysis

The Kolmogorov Smirnov test was used to test for normality. A correlation between ligament dimensions and the TCA was performed using Pearson’s correlation coefficient. Differences between sex, side, and groups of single and double fascicular ligament were considered statistically significant if the *p*-value was less than .05. Repeated measures (test–retest) reliability analyses via interclass correlation coefficients (ICC) (ICC(2,1) and ICC(2,2)) and 95% confidence intervals were obtained. ICC values were interpretated according to Fleiss et al. [17], where a value of 0.75 or greater indicates excellent reliability, 0.40 to 0.75 indicates fair to good reliability, and 0.40 or less indicates poor reliability. The standard error of the measurement (SEM) was calculated to determine the absolute between-trial variability in scores [18]. The data were analyzed using SPSS Statistics software version 22.0. The Swedish Ethical Review Authority approved the study and waived the need for informed consent (number 2020–06177). Our study was performed in accordance with relevant named guidelines and regulations.

## Results

### The anterior talofibular ligament

**Fig. 4:**
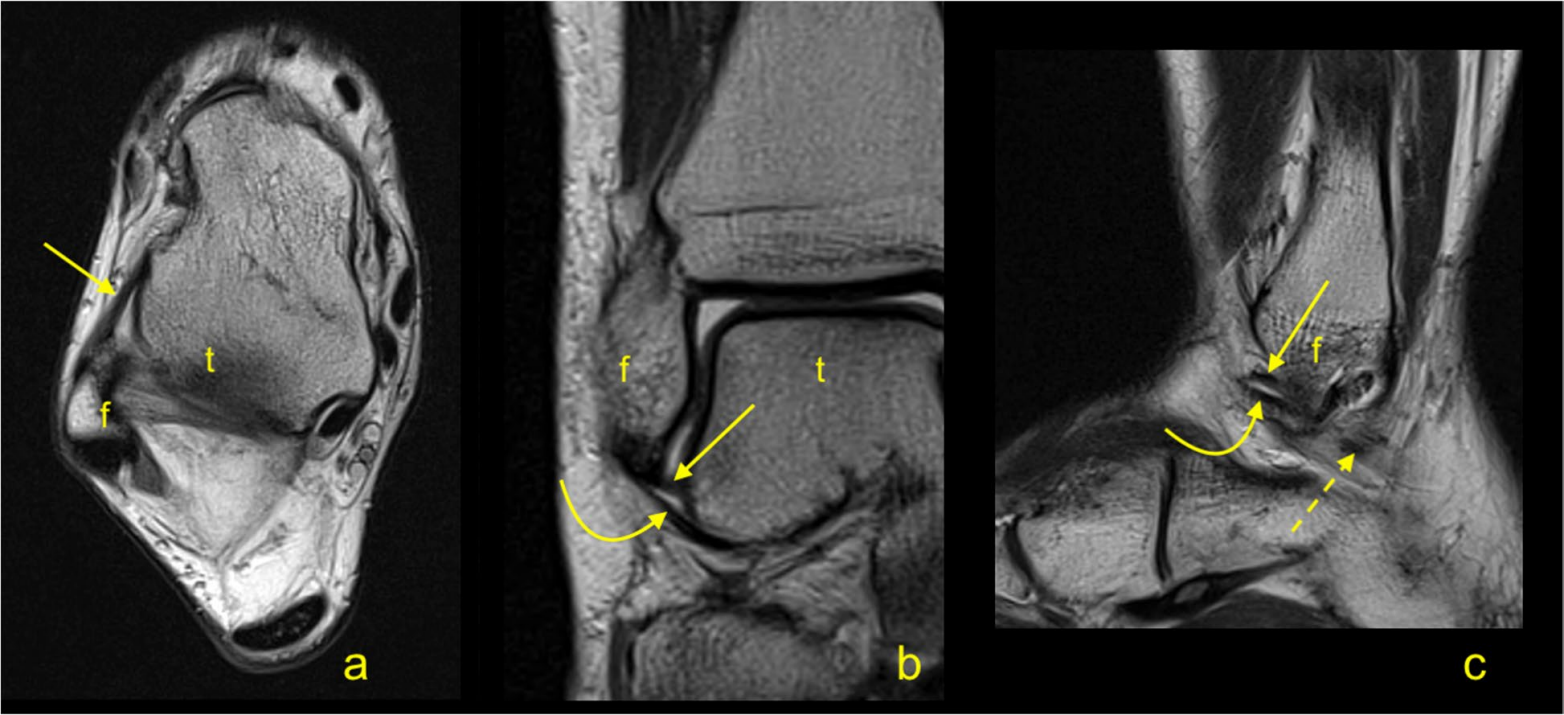
A double fascicular anterior talofibular ligament. Two asymmetrical fascicles are visible; the bigger one is located in a superior position (arrow), and the smaller one inferior (curved arrow). **A** T2-weighted sagittal section; **b** and **c** T2-weighted axial section; **d** and **e** T2-weighted coronal section. F, fibula; t, talus

**Fig. 5:**
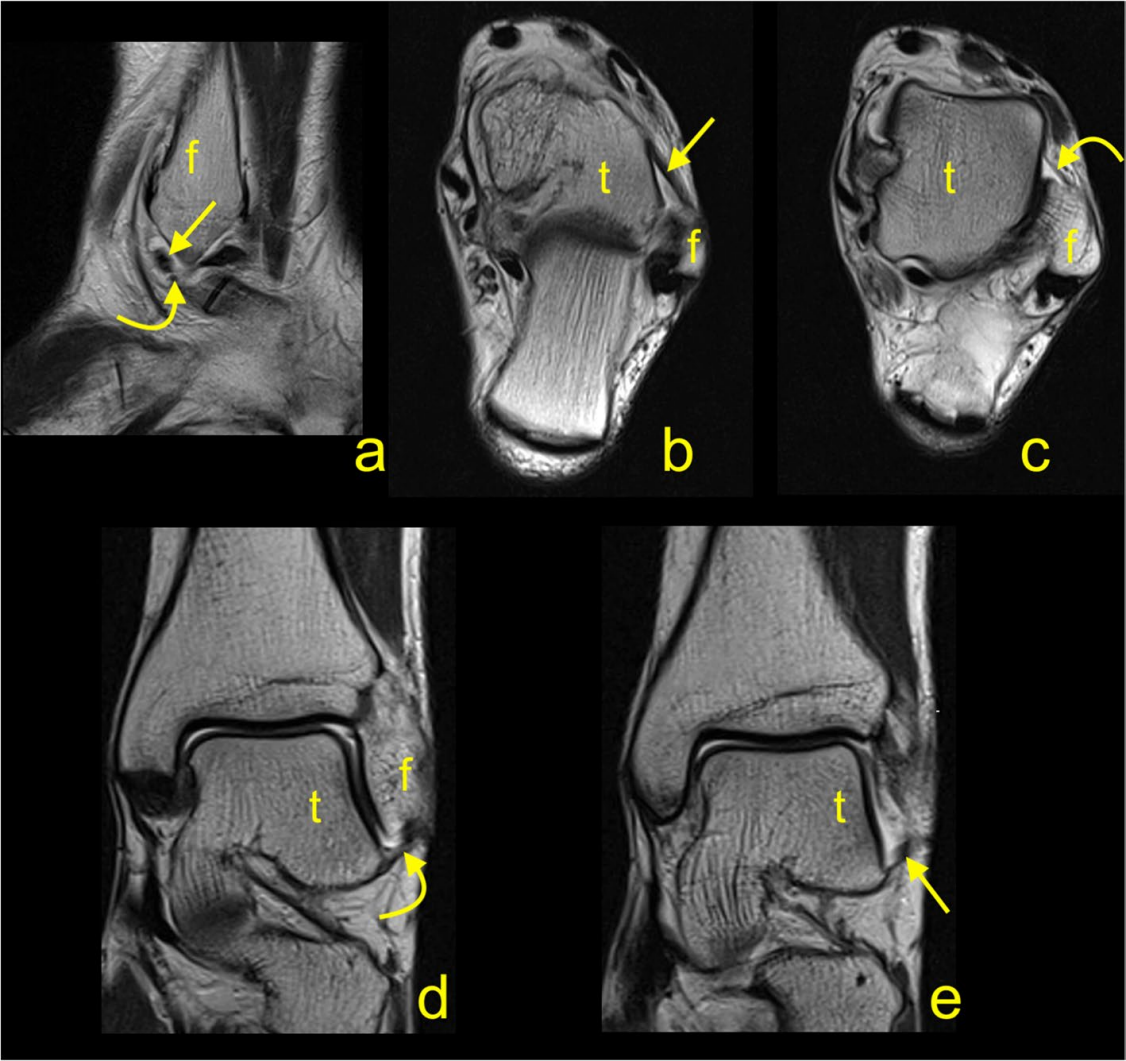
A double fascicular anterior talofibular ligament. Two symmetrical fascicles are visible: a superior fascicle (arrow) and an inferior fascicle (curved arrow). The dashed arrow shows the midportion of the CFL. **A** T2-weighted axial section; **b** T2-weighted coronal section; **c** T2-weighted sagittal section. F, fibula; t, talus

The intra-observer agreement was measured by ICC values which ranged from 0.58 to 0.89 for individual measurements (Table 1) while the inter-observer agreement ranged from 0.41 to 0.70 (Table 2). The SEM values of the individual measurements are provided in Table 1. We identified a group of double fascicular ATFL (77%, *n* = 193) (Figs. 4 and 5) and a group of single fascicular ATFL (23%, *n* = 58). No other variations were identified. The average length of the ATFL was 24.5 ± 3.3 mm, and no significant differences were found between single and double fascicular ATFL (Table 1). The average thickness of the ATFL was 2.2 ± 0.3 mm, with a statistically significant difference between groups. In single fascicular ATFL, the average thickness was 2.6 ± 0.4 mm, while in double fascicular ATFL, this was 2.1 ± 0.2 mm (Table 1). The average width of the ATFL was 5 ± 0.7 mm, but no statistically significant differences were noted between groups of single and double fascicular ligaments (Table 1). The length of the fibular attachment was longer than that of the talar attachment, which was, on average, 5.7 ± 0.9 mm, with statistically significant differences between groups (*p* < 0.001). No statistically significant differences were found regarding the length of the talar attachment. The average volume of the ATFL was 250.9 mm^3^, which corresponds to 0.3 mL, with statistically significant differences between groups (*p* < 0.001), as shown in Table 1. There were no statistically significant differences between the ATFL dimensions obtained on the 1.5 T and 3.0 T machines (*p* > .05).

**Table 1:** Dimensions of the anterior talofibular ligament in the general group, single fascicular and double fascicular ligaments

		length [mm]	thickness [mm]	width [mm]	fibula [mm]	talus [mm]	volume [mm^3^]
General *N* = 251	average	24.5	2.2	5.0	5.7	4.3	250.9
	SD	3.3	0.3	0.7	0.9	0.5	50.9
	min	16.1	1.3	3.4	3.7	3.1	93.3
	max	33.1	3.0	6.9	7.3	5.6	375.7
		length [mm]	thickness [mm]	width [mm]	fibula [mm]	talus [mm]	volume [mm^3^]
single fascicular *n* = 58	average	24.2	2.6	4.9	6.2	4.2	297.5
	SD	2.8	0.4	0.7	0.7	0.6	47.2
	min	19.3	1.9	4.5	4.8	3.5	187
	max	27.9	3.0	6.9	6.9	5.2	375
		length [mm]	thickness [mm]	width [mm]	fibula [mm]	talus [mm]	volume [mm^3^]
double fascicular *n* = 193	average	24.6	2.1	5.0	5.6	4.3	257.5
	SD	3.4	0.2	0.7	0.9	0.5	50.2
	min	16.1	1.3	3.4	3.7	3.1	93.3
	max	33.1	2.8	6.7	7.3	5.6	375.7
*p*		> 0.05	< 0.001	> 0.05	< 0.001	> 0.05	< 0.001
ICC (95% CI)		0.89 (0.88–0.89)	0.82 (0.83–0.86)	0.81 (0.67–0.86)	0.58 (0.56–0.69)	0.60 (0.59–0.76)	0.74 (0.63–0.88)
SEM		0.83	0.08	0.12	0.17	0.08	19.1

Abbreviations: *ICC* intra-class correlation coefficient, *min.* Minimum, *max.* Maximum, *SEM* standard error of measurement, *SD* standard deviation. *P*-value refers to difference between single and double fascicular ligament

**Table 2:** The Inter-observer agreement

ATFL	length [mm]	thickness [mm]	width [mm]	fibula [mm]	talus [mm]	volume [mm^3^]
ICC	0,7	0,60	0,49	0,46	0,41	0,58
	(0,19 - 0,91)	(0,00 - 0,88)	(−0,07 - 0,83)	(−0,23 - 0,84)	(−0,13 - 0,80)	(0,03 – 0,87)
CFL	length [mm]	thickness [mm]	width [mm]	fibula [mm]	calcaneus [mm]	volume [mm^3^]
ICC	0,68	0,77	0,49	0,63	0,69	0,47
	(0,17 - 0,91)	(0,34 - 0,94)	(−0,22 - 0,85)	(0,01 - 0,90)	(0,13 - 0,91)	(0,17 – 0,83)

*ATFL* the anterior talofibular ligament, *CFL* the calcaneofibular ligament, *ICC* intra-class correlation coefficient

**Fig. 6:**
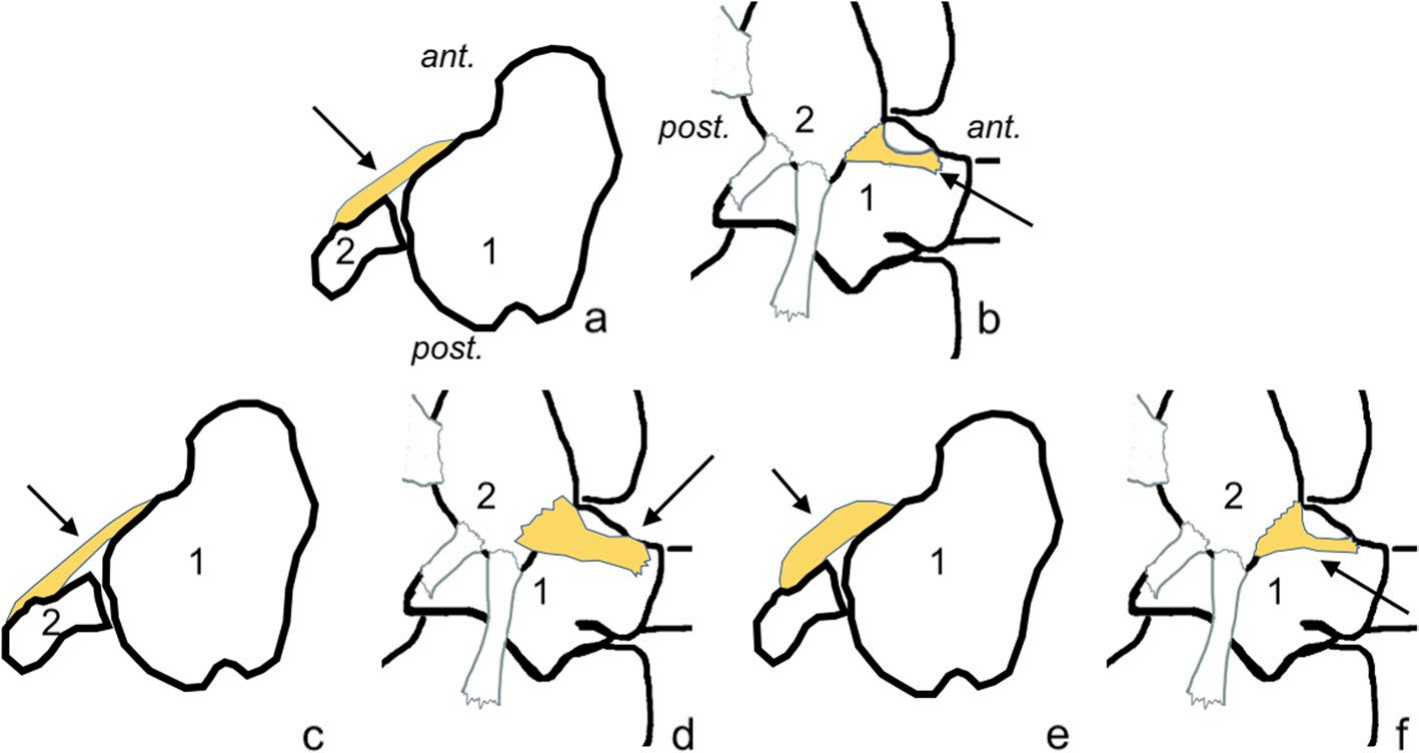
Examples of correlations between the dimensions of the anterior talofibular ligament. **A**, **c**, and **e** transverse section; **b**, **d**, and **f** view from the lateral side. Ant., anterior; post., posterior; 1, talus; 2, fibula. **C** and **d** the increased length of the anterior talofibular ligament is positively correlated with width and negatively correlated with thickness. **E** and **f** the increased thickness of the ATFL is positively correlated with the length of the fibular insertion and negatively correlated with the width and length of the talar insertion, and there is a very weak positive correlation with the length of the whole ligament

Our study revealed very weak but significant negative correlations between the length and thickness in all groups (Table 3, Fig. 6). There was a significant positive correlation between the width and length, but there was a negative correlation between the width and the thickness. This tendency was most prominent in single fascicular ATFL.

**Table 3:** Correlation of the anterior talofibular ligament’s dimensions

		length	thickness	width	fibular	talar	volume
General *N* = 252	length	x	−0.02***	0.20***	0.15***	0.05***	0.67***
	thickness		x	−0.13***	0.21***	−0.15***	0.45***
	width			x	−0.02**	0.49***	0.62***
	fibula				x	0.05***	0.17***
	talus					x	0.20***
	volume						x
Single fascicular *n* = 58	length	x	− 0.01***	0.39***	−0.07***	0.01***	0.86***
	thickness		x	− 0.49***	−0.19***	− 0.60***	0.43***
	width			x	0.01*	−0.04	0.45***
	fibula				x	−0.08***	− 0.16***
	talus					x	−0.4***
	volume						x
Double fascicular *n* = 193	length	x	−0.003***	0.14***	0.25***	0.06***	0.65***
	thickness		x	−0.01***	0.27***	0.05***	0.44***
	width			x	−0.03*	0.67***	0.68***
	fibula				x	0.03***	0.43***
	talus					x	0.43***
	volume						x

* *p* < 0.05, ** *p* < 0.01, *** *p* < 0.001

Variable correlation could be seen between the dimensions of the bone attachments and the other diameters. A negative correlation between the length and the thickness of the ATFL and the length of the fibular attachment in single fascicular ATFL was found (Table 3). The length of the talar attachment of the ATFL showed a negative correlation with the thickness of single fascicular ATFL. The length of the talar attachment in double fascicular ATFL showed a strong positive correlation with the width. The volume of the ATFL correlated with all diameters, however, mostly with length (Table 1).

### The calcaneofibular ligament

The inter-rater ICC values ranged from 0.66 to 0.78 for individual measurements (Table 4), while the inter-observer agreement ranged from 0.47 to 0.77 (Table 2). The SEM values of the individual measurements are provided in Table 4. A group of double fascicular (69.7%, *n* = 175) (Fig. 6) and single fascicular (30.3%, *n* = 76) CFLs was identified. The average length of the CFL was 33.7 ± 4.1 mm. Statistically significant differences (*p* < 0.001) between the two groups were found (Table 4). The average thickness of the CFL was 2.1 ± 0.2 mm; however, the differences between groups were not significant. The average width was 6.6 ± 1.4 mm, without a significant difference between groups. Significant differences (*p* < 0.001) in volume were revealed, with an average value of 419.8 mm^3^. The average craniocaudal dimension of the calcaneal attachment of the CFL was 7.7 ± 1.3 mm, with significant difference between groups (*p* < 0.01) (Table 5). There were no statistically significant differences between the CFL dimensions obtained on the 1.5 T and 3.0 T machines (*p* > .05).

**Table 4:** Dimensions of the calcaneofibular ligament in the general group, single fascicular and double fascicular ligaments

		length [mm]	thickness [mm]	width [mm]	fibula [mm]	calcaneus [mm]	volume [mm^3^]	CFL angle [deg]	TCA [deg]
General *N* = 251	average	33.7	2.1	6.6	5.2	7.7	419.8	113.5	10.9
	SD	4.1	0.2	1.4	1.0	1.3	98.2	13.1	4.5
	min	24.2	1.3	3.8	2.7	4.9	248.8	80.2	2.2
	max	42.7	2.5	10.1	7.1	10.1	656.6	133.4	16.7
		length [mm]	thickness [mm]	width [mm]	fibula [mm]	calcaneus [mm]	volume [mm^3^]	CFL angle [deg]	TCA [deg]
Single fascicular *n* = 76	average	31.4	2.0	6.2	5.4	7.1	383.8	116.1	12.4
	SD	3.5	0.1	1.7	0.9	1.3	99.0	12.6	5.3
	min	24.7	1.8	3.8	3.1	4.9	248.8	90.0	2.0
	max	42.7	2.1	9.2	6.4	8.9	290.3	133.0	23.0
		length [mm]	thickness [mm]	width [mm]	fibula [mm]	calcaneus [mm]	volume [mm^3^]	CFL angle [deg]	TCA [deg]
double fascicular *n* = 175	average	33.2	2.1	6.5	5.2	7.6	437.0	113.1	10.3
	SD	4.3	0.3	1.3	1.1	1.3	93.7	13.2	3.8
	min.	24.2	1.3	4.1	2.7	5.1	277.2	80.0	3.0
	max	42.7	2.5	10.1	7.1	10.1	656.6	140.0	17.0
	P	< 0.001	> 0.05	> 0.05	> 0.05	< 0.01	< 0.001	> 0.05	< 0.05
ICC (95% CI)		0.78 (0.74–0.79)	0.66 (0.39–0.75)	0.78 (0.66–0.89)	0.74 (0.63–0.88)	0.72 (0.60–0.77)	0.74 (0.67–0.78)	0.84 (0.73–0.88)	0.83 (0.34–0.95)
SEM		0.90	0.06	0.24	0.21	0.18	27.8	2.98	0.48

Abbreviations: *CFL* the calcaneofibular ligament, *deg.* degrees, *ICC* intra-class correlation coefficient, *min.* Minimum, *max.* Maximum, *SEM* standard error of measurement, *SD* standard deviation, *TCA* the tibiocalcaneal angle. *P*-value refers to difference between single and double fascicular ligament

**Table 5:** Correlations of the calcaneofibular ligament’s dimensions

		length	thickness	width	fibula	calcaneus	volume	CFL angle	TCA
General *N* = 251	length	x	- 0.19***	- 0.13***	- 0.23***	- 0.09***	0.22***	−0.44***	0.13***
	thickness		x	- 0.18***	- 0.17***	−0.08***	0.21***	0.26***	−0.23***
	width			x	0.48***	0.56*	0.73***	0.33**	−0.20***
	fibula				x	0.56***	0.21***	0.21***	0.01***
	calcaneus					x	0.57***	0.22***	−0.27***
	volume						x	0.25***	−0.28***
Single fascicular *n* = 76	length	x	- 0.51***	- 0.16***	- 0.42***	−0.02***	0.01***	−.67***	0.19***
	thickness		x	- 0.10***	0.4***	0.14***	0.06***	0.13***	0.13***
	width			x	0.51	0.57	0.51	0.42***	−0.29**
	fibula				x	0.56**	0.52***	0.29***	0.12***
	calcaneus					x	0.78***	0.24**	−0.31***
	volume						x	0.16***	−0.21***
Double fascicular *n* = 175	length	x	- 0.03***	- 0.11***	- 0.13***	- 0.12***	0.21***	−0.22***	0.17***
	thickness		x	- 0.24***	- 0.33***	−0.2***	0.29***	0.36***	−0.44***
	width			x	0.47**	0.52*	0.47*	0.29***	−0.09***
	fibula				x	0.58***	0.13***	0.20***	−0.10*
	calcaneus					x	0.44***	0.26***	−0.23***
	volume						x	0.41***	−0.32***

Abbreviations: *CFL* the calcaneofibular ligament, *TCA* the tibiocalcaneal angle. * *p* < 0.05, ** *p* < 0.01, *** *p* < 0.001

The inter-rater ICC value for the CFL angle was 0.84 (0.73–0.88, SEM 2.98) (Table 4), while the inter-observer agreement was 0.43 (0.34–0.65). The average CFL angle was 113.5 ± 13.1 deg, with no significant differences between groups (Table 5). The inter-rater ICC value for the TCA was 0.83 (0.34–0.95, SEM 0.48) (Table 4), while the inter-observer agreement was 0.39 (0.32–0.52). The average value of the TCA was 10.9 ± 4.5 deg, with significant differences between groups (Tables 4 and 5). There were no statistically significant differences between CFL or TCA angles obtained on the 1.5 T and 3.0 T machines (*p* > .05).

**Fig. 7:**
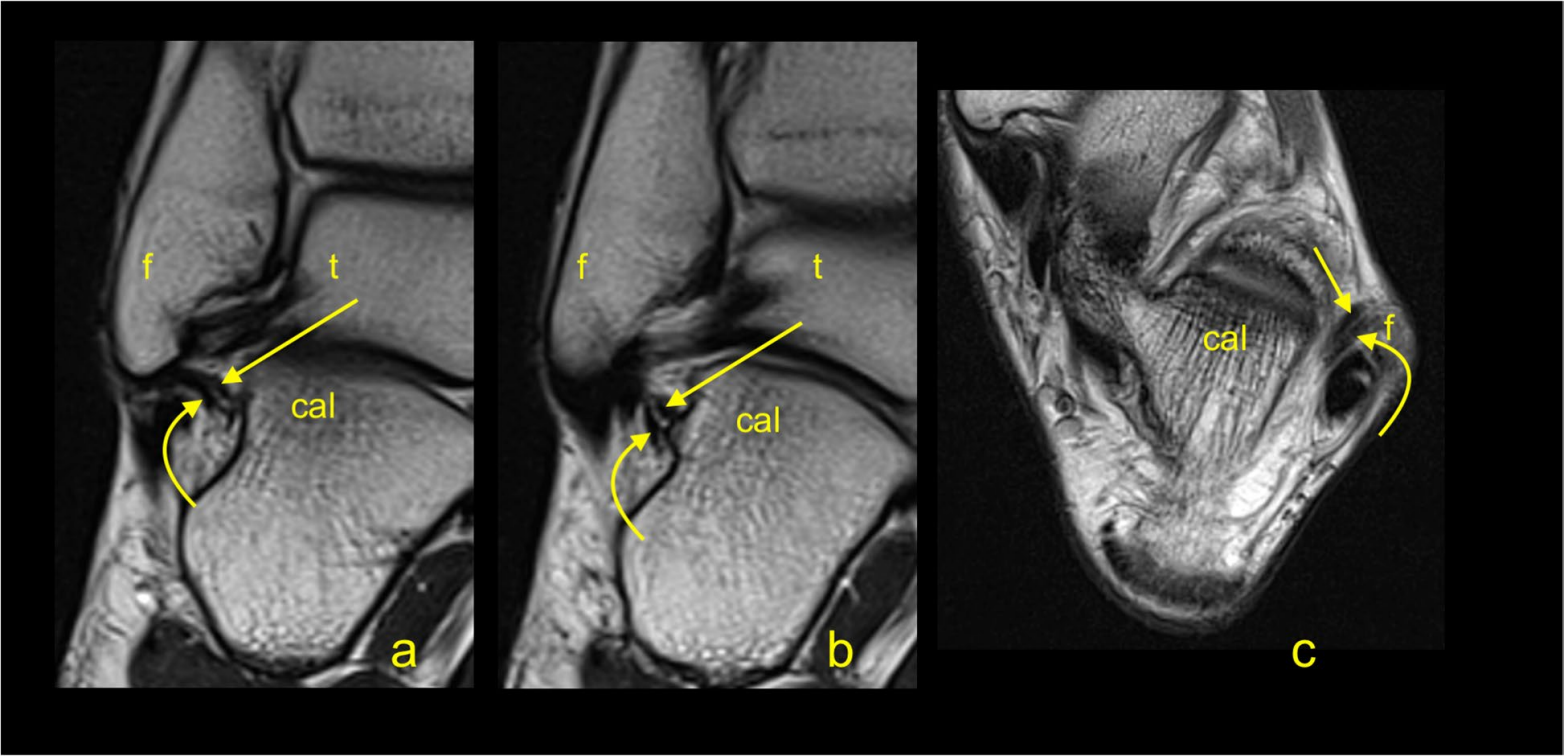
The double fascicular calcaneofibular ligament. The medial (arrow) and lateral fascicle (curved arrow) are visible. **A** and **b** T2-weighted coronal section; **c** T2-weighted axial section. Cal, calcaneus; f, fibula; t, talus

**Fig. 8:**
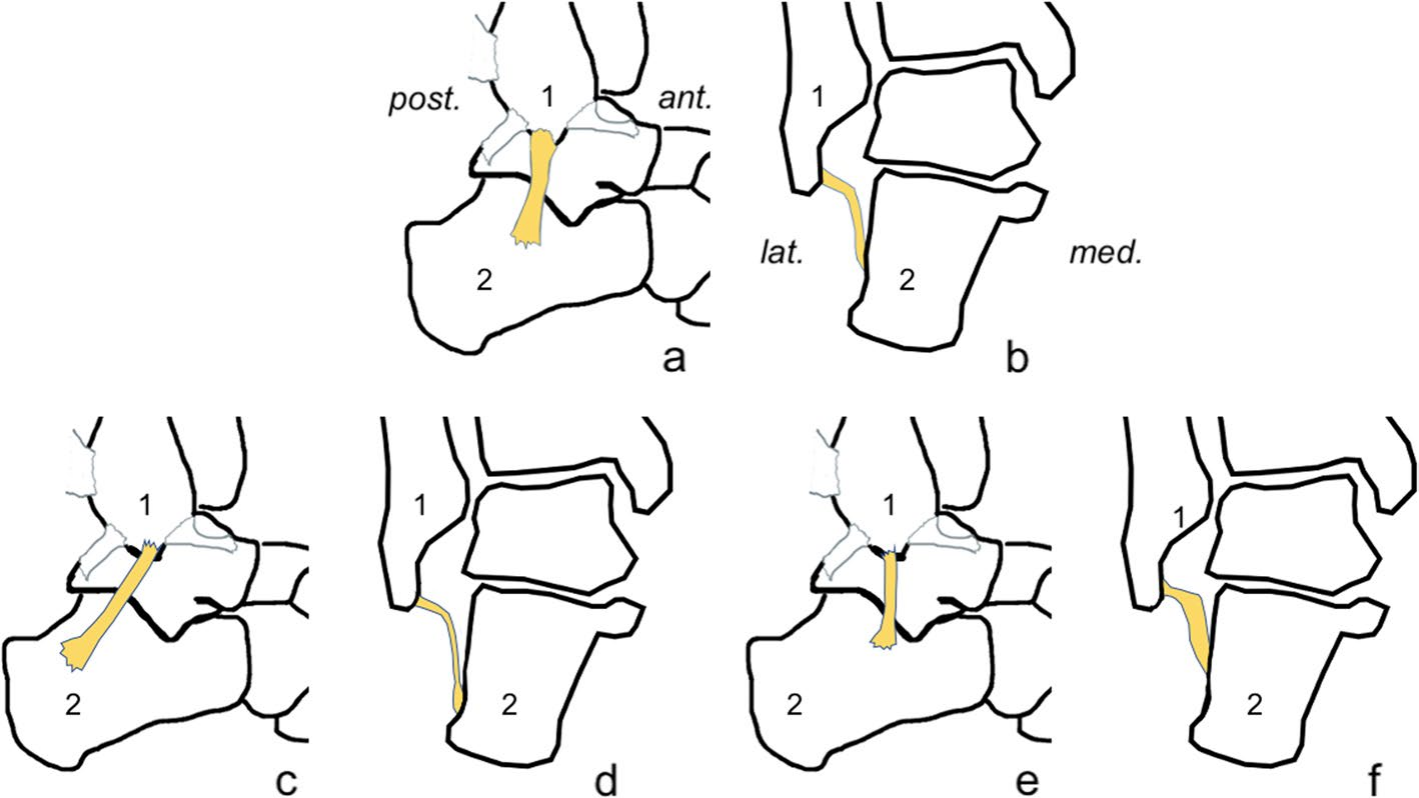
Examples of correlations between the dimensions of the calcaneofibular ligament. **A**, **c**, and **e** lateral view; **b**, **d**, and **f** coronal section. Ant., anterior; post., posterior; med., medial; lat., lateral. **C** and **d** the increased length is negatively correlated with the width, thickness, and length of the fibular attachment and the CFL angle. **E** and **f** the increased thickness is positively correlated with the CFL angle and negatively correlated with the length of the fibular insertion and the width and length of the whole ligament, and there is a very weak negative correlation with length of the calcaneal attachment

We revealed some statistically significant correlations between the dimensions of the CFL (Tables 4 and 5, Figs. 7 and 8). Significant negative correlations were found between the length, thickness, and width. The most prominent negative correlation between the length and thickness was seen in single fascicular CFL. Significant positive correlations between the volume and all dimensions were noted, with the strongest correlation with the width; however, these were not significant in single fascicular CFL (Table 5).

The width of the fibular attachment was correlated with CFL length, thickness, and width (Table 5). Width was positively correlated with the length of bony attachments, with significance only in double fascicular CFL (Table 5). A significant positive correlation between the dimensions of both bony attachments was noted (Table 5). The strongest significant positive correlation was between volume and width in double fascicular CFL (Table 5, Fig. 8). A significantly negative correlation between the CFL angle and the length of the CFL was seen, mainly in the group of single fascicular ligaments. The TCA was negatively correlated with thickness, which was most prominent in double fascicular CFL.

No statistically significant differences between sex, side, and groups of single and double fascicular ligaments or dimensions were found (*p* > .05). We noted two cases of os subfibulare, where the inferior fascicle of the ATFL had an insertion on the accessory bone and a superior fascicle on the fibula.

## Discussion

Our study revealed the negative correlation between thickness and width of the ATFL and the positive correlation between thickness and length. The negative correlation was found between length and width for the CFL. We found significant differences in dimensions between single and double fascicular ATFL and CFL Significant differences in the midportion thickness, the fibular attachment length, and the volume between single and double fascicular ATFL can be noticed. In the CFL, significant differences were noted in the length of the whole ligament, the length of the calcaneus attachment, and the volume.

The ligament dimensions may be clinically relevant because the possibility of injury is more common when the ligament is wider and longer. More anatomical reconstruction helps to restore the ligament’s native geometry and thus helps to restore joint stability [19]. The intercorrelation between the length, the width, and the thickness of the ATFL and the CFL in relation to fascicular structures has not been studied before. The ATFL should not be understood as a single ligament but rather as a component of a biomechanical complex. This complex, in different positions of the joint and in different directions of force, tenses in different ways, and its extended parts act as stabilizers for post-secondary movements.

Some differences from previously published results were noted in the dimensions of the ATFL. This is probably related to the different methodology and the smaller groups in previous studies. The length of the ATFL obtained by us was about 10 mm longer than values reported previously on a smaller number of cases [7, 8, 20–22]. However, when compared with authors who conducted studies on more extensive sample, our results are comparable despite the different methodology [4, 6] (Tables 1 and 3). The reason for our finding of a longer ATFL than other authors may be due to the fact that we measured the entire ligament, including its bony attachments, while some authors measured from the midpoint of the attachment. It is also possible that MRI can better differentiate the structure of the ligament from its bony attachment [7, 23]. In future research, the methodology of the research on ankle ligaments should be standardized. From a clinical point of view, it is better to use our method because it refers to a functional ligament complex, which the surgeon also sees during the procedure. The dimensions of the ATFL may have a developmental context because it is significantly wider in clubfoot than in the general population [24, 25].

The thickness of the ATFL and the CFL has not been studied as much as the length or width. The focus on length and width is related to the fact that these dimensions are most relevant for orthopedists in the context of surgical reconstruction. The normal thickness of a structure has importance to the radiologist during the evaluation of MRI after trauma and chronic injuries. A partial injury to a ligament may preserve a narrow ligamentous band that has no mechanical properties or may affect only a small part of the ligament, which may still have good function. Both are anatomically classified as grade two injuries; although, they are functionally very different. The amount of ligament defect after trauma should be specified in the radiological report. Thus, the thickness of the torn ligament at grade two should be compared to a normal cut-off value. However, this value is not specified in the literature. Our results are comparable to the sparse papers available [6, 26, 27]. In our opinion, the use of MRI enables an accurate assessment of the degree of ligament injury, which allows for planning possible surgical treatment.

No significant differences were noted between the thickness of the CFL revealed by us compared with previously reported values [9]. As with the ATFL, the length and width of the CFL revealed by us differed from the previously published results of anatomical studies on cadavers. The difference in the length of the CFL was about 10 mm, and the difference in the width was about 4 mm [5, 6, 8]. The differences probably arise from different methodologies and the smaller number of cases in previously published studies.

A CFL angle lower than 119 deg measured on MRI may indicate valgus hindfoot [28]. This is due to the fact that a decreased CFL angle is associated with an increased TCA, which may correspond to valgus deformity [28]. Quite prominent correlations were visible between CFL diameters, CFL angle, and TCA values. The orientation of the CFL in relation to its thickness can be a valuable factor during surgical reconstruction. A confirmation of this opinion can be the observation that a horizontally orientated CFL angle may indicate hindfoot [28]. Conventionally, the hindfoot assessment has been performed on standardized X-ray, and values of more than 10 deg are considered abnormal [29]. The values obtained by us are somewhat difficult to compare with the values reported on X-ray because the patient’s position during MRI and X-ray is different [28, 29]. However, there is a correlation between CFL angle and the TCA [28]. This is essential to avoid possible overloading of the reconstructed CFL by hindfoot deformation.

We acknowledge the limitations of our study. Only the longest dimension of the bony attachments was measured. We believe that a special protocol with ultra-thin layers or 3D sequences should be used to improve measurement accuracy. It will enable reformatting at the plane along or perpendicular to the measured ligament. Measurement errors may be related to slide thickness and the angle of the section. The retrospective character and the lack of surgical correlation may also be limitations of this study. Another limitation is the lack of information about the subjects’ height, weight, and body mass index (BMI). Only one operator performed the measurements. MRI examinations were performed on two different MRI machines.

## Conclusion

Most dimensions of the ATFL and the CFL correlated with each other. There are significant differences in dimensions between single and double fascicular ligaments. Routine MRI may be used to assess ATFL and CFL correlations of dimensions. The data from this study may be used for the development of a virtual biomechanical model of the human foot.

## Data Availability

The datasets used and/or analyzed during the current study are available from the corresponding author upon reasonable request.

## Abbreviations

ATFL: Anterior talofibular ligament
BMI: Body Mass Index
CFL: Calcaneofibular ligament
FOV: Field-of-view
ICC: Intraclass correlation coefficient
MRI: Magnetic resonance imaging
PD: Proton density
SEM: Standard error of the measurement
STIR: Short-T1 inversion recovery
T1-TSE: T1-weighted turbo spin echo
TCA: The tibiocalcaneal angle
TE: Echo time
TR: Repetition time
TSE: Turbo spin echo

## References

[1] Chen C, Lu H, Hu J, Qiu X, Li X, Sun D (2018). Anatomic reconstruction of anterior talofibular ligament with tibial tuberosity-patellar tendon autograft for chronic lateral ankle instability. J Orthop Surg (Hong Kong).

[2] Higashiyama R, Sekiguchi H, Takata K, Katagiri A, Inoue G, Takaso M (2020). Anatomical arthroscopic anterior Talofibular ligament repair and reconstruction using a free tendon. Arthrosc Tech.

[3] Langner I, Frank M, Kuehn JP, Hinz P, Ekkernkamp A, Hosten N (2011). Acute inversion injury of the ankle without radiological abnormalities: assessment with high-field MR imaging and correlation of findings with clinical outcome. Skelet Radiol.

[4] Edama M, Kageyama I, Kikumoto T, Nakamura M, Ito W, Nakamura E (2018). Morphological features of the anterior talofibular ligament by the number of fiber bundles. Ann Anat.

[5] Kobayashi T, Suzuki D, Kondo Y, Tokita R, Katayose M, Matsumura H (2020). Morphological characteristics of the lateral ankle ligament complex. Surg Radiol Anat.

[6] Taser F, Shafiq Q, Ebraheim NA (2006). Anatomy of lateral ankle ligaments and their relationship to bony landmarks. Surg Radiol Anat.

[7] Wenny R, Duscher D, Meytap E, Weninger P, Hirtler L (2015). Dimensions and attachments of the ankle ligaments: evaluation for ligament reconstruction. Anat Sci Int.

[8] Yıldız S, Yalcın B (2013). The anterior talofibular and calcaneofibular ligaments: an anatomic study. Surg Radiol Anat.

[9] Dimmick S, Kennedy D, Daunt N (2008). Evaluation of thickness and appearance of anterior talofibular and calcaneofibular ligaments in normal versus abnormal ankles with MRI. J Med Imaging Radiat Oncol.

[10] Higashiyama R, Aikawa J, Iwase D, Takamori Y, Watanabe E, Takaso M (2019). Anatomical arthroscopic anterior Talofibular ligament and Calcaneofibular ligament reconstruction using an autogenic hamstring tendon: safe creation of anatomical fibular tunnel. Arthrosc Tech.

[11] Kennedy JG, Smyth NA, Fansa AM, Murawski CD (2012). Anatomic lateral ligament reconstruction in the ankle: a hybrid technique in the athletic population. Am J Sports Med.

[12] Drakonaki E, Gataa K, Solidakis N, Szaro P (2021). Anatomical variations and interconnections of the superior peroneal retinaculum to adjacent lateral ankle structures: a preliminary imaging anatomy study. J Ultrason.

[13] Dalmau-Pastor M, Malagelada F, Calder J, Manzanares MC, Vega J (2020). The lateral ankle ligaments are interconnected: the medial connecting fibres between the anterior talofibular, calcaneofibular and posterior talofibular ligaments. Knee Surg Sports Traumatol Arthrosc.

[14] Szaro P, Ghali Gataa K, Polaczek M, Ciszek B (2020). The double fascicular variations of the anterior talofibular ligament and the calcaneofibular ligament correlate with interconnections between lateral ankle structures revealed on magnetic resonance imaging. Sci Rep.

[15] Edama M, Takeishi M, Kurata S, Kikumoto T, Takabayashi T, Hirabayashi R (2019). Morphological features of the inferior fascicle of the anterior inferior tibiofibular ligament. Sci Rep.

[16] Buck FM, Hoffmann A, Mamisch-Saupe N, Farshad M, Resnick D, Espinosa N (2013). Diagnostic performance of MRI measurements to assess hindfoot malalignment. An assessment of four measurement techniques. Eur Radiol.

[17] Fleiss JL (2011) Design and analysis of clinical experiments. United States: Wiley

[18] Portney LG, Watkins MP (2009) Foundations of Clinical Research: Applications to Practice. Prentice Hall

[19] Akseki D, Pinar H, Yaldiz K, Akseki NG, Arman C (2002). The anterior inferior tibiofibular ligament and talar impingement: a cadaveric study. Knee Surg Sports Traumatol Arthrosc.

[20] Khawaji B, Soames R (2015). The anterior talofibular ligament: a detailed morphological study. Foot (Edinb).

[21] Milner CE, Soames RW (1997). Anatomical variations of the anterior talofibular ligament of the human ankle joint. J Anat.

[22] Raheem OA, O'Brien M (2011). Anatomical review of the lateral collateral ligaments of the ankle: a cadaveric study. Anat Sci Int.

[23] Kristen KH, Seilern Und Aspang J, Wiedemann J, Hartenbach F, Platzgummer H (2019). Reliability of ultrasonography measurement of the anterior talofibular ligament (ATFL) length in healthy subjects (in vivo), based on examiner experience and patient positioning. J Exp Orthop.

[24] Uchiyama E, Kim JH, Abe H, Cho BH, Rodriguez-Vazquez JF, Murakami G (2014). Fetal development of ligaments around the tarsal bones with special reference to contribution of muscles. Clin Anat.

[25] Windisch G, Anderhuber F, Haldi-Brandle V, Exner GU (2007). Anatomical study for an updated comprehension of clubfoot. Part II: ligaments, tendons and muscles. J Child Orthop.

[26] Burks RT, Morgan J (1994). Anatomy of the lateral ankle ligaments. Am J Sports Med.

[27] Kanamoto T, Shiozaki Y, Tanaka Y, Yonetani Y, Horibe S (2014). The use of MRI in pre-operative evaluation of anterior talofibular ligament in chronic ankle instability. Bone Joint Res.

[28] Lee S, Oliveira I, Pressney I, Welck M, Saifuddin A (2020). The horizontal calcaneofibular ligament: a sign of hindfoot valgus on ankle MRI. Skelet Radiol.

[29] Reilingh ML, Beimers L, Tuijthof GJ, Stufkens SA, Maas M, van Dijk CN (2010). Measuring hindfoot alignment radiographically: the long axial view is more reliable than the hindfoot alignment view. Skelet Radiol.

